# Case of Menorrhagia

**Published:** 1840-07-11

**Authors:** A. D. Chaloner

**Affiliations:** Philadelphia


### CASE OF MENORRHAGIA

*Of seven weeks' duration, cured by the Extract of Monesia.* Reported by A. D. CHALONER, A. M., MD.

On *Friday, July 2d,* at 2½ o'clock, P. M., I was called to see Mrs. L——rs, æt. 35, of sanguine temperament, who was suffering under a severe attack of menorrhagia, of *seven weeks' duration.* A month previous to the attack, she had miscarried, (three and a half months advanced in pregnancy,) and recovered under the usual course of treatment; but having over exerted her strength, and taken cold, she was attacked with a profuse "flow of blood from her womb," at first mistaken for the return of the catamenia. It increased, however, gradually, and she had recourse, previously to my visit, to various cold and astringent injections, in connexion with the elixir vitriol. These remedies failing, and the discharge increasing, she became alarmed and sent for me. I found her, on my arrival, sitting up in a chair, countenance pale and anxious, pulse slow, violent pains in lumbar and sacral region, and the slightest motion causing a profuse discharge. She was immediately placed in her bed, ordered to take *cold* drinks, and the following recipe: ℞. Acetat. Plumbi, ℈jss., G. Opii, gr. iv. M. ft. pil. No. xii. S. one every hour, and an injection per vaginam of ℞., Sulphat. Zinci, gr. viii., Aq. Font. f℥i.—her feet and legs were elevated by means of blocks of wood placed beneath the feet of the bedstead, and *perfect rest* enjoined.

*July 3d.* Had slept some, pulse more natural, skin cool, the discharge undiminished.

*Treatment*—Increase the acetas plumbi, &c. to 8 grs. every hour, in conjunction with a third of a grain of opium; continue injections; ice in a bladder to be applied over the pubic region.

*Vesper.* Feels better; a perceptible diminution of the discharge; treatment to be continued as before.

*July 4th.* Having been prevented from seeing Mrs. L——rs to-day, my friend, Dr. N. Benedict, saw her for me, and finding the hæmorrhage returning, and bowels constipated, gave her the following ℞. Secale Cornutum, ʒi., Sacch. Albi., G. Acaciæ, q. s., Aq. Font. f℥vi., M. S. a table-spoonful every four hours,—also, these pills: ℞. Hieræ Picræ, ʒss., Sapo. Venet. gr. iv., Syrup. Rhei, q. s., M. ft. pil. x., S. two every night.

*July 5th.* Bowels opened; discharge still continues, and profuse, and increased by motion; treatment continued.

*July 6th.* Returned to the city and saw her with Dr. B. No abatement of the discharge. Treatment—to take of the infusion of secale cornutum a table-spoonful every hour; elixir vitriol, 30 gtt. in "eau sucré," at the same time.

*Vesper.* Discharge continues; feels very weak. Treatment—℞. Secale Cornutum Pulv. Sacch. Alb. āā ℈ij, M. ft. pulv. No. viii., S. one every half hour until pains are produced.

*July 7th.* Has taken *seventy grains* of ergot without causing uterine pains, or affecting the discharge. Treatment—℞. Acet. Plumbi ℈jss., Tinct. Opii fʒj., Aq. Font. f℥ij., ft. enema, S. one-half at once, the remainder in an hour; elixir vitriol, as a tonic, iced drinks, astringent injections per vaginam, warming plaster to sacral region.

*July 8th.* The uterus, on examination per vaginam, was soft and spongy; in the commencement of the treatment it was slightly swollen, and the anterior lip enlarged; pain in the back and the discharge continue. Treatment—℞. Tinct. Cinnamon f℥ij., S. thirty drops every hour; ℞. Prussiat. Ferri, ʒj., G. Aloes, gr. v., Conserv. Rosav. q. s., M. et divid. in pil. xx. S. one four times daily; alum water injections, a compress of folded napkins, and a broad roller applied tightly over the uterine region.

*Vesper.* Feels better; bowels opened freely; to take but two pills and tinct. cinnamon, as before; discharge as before.

*July 9th.* The discharge having increased in frequency and quantity, I procured twelve pills of the EXTRACT OF MONESIA,* of three grains each, and at 2 o'clock, P. M. gave her six pills—*one* to be taken every *hour and a half,* until they had an effect upon the discharge.

*Vesper.* Has taken but three pills, (3 grs. each;) the first pill having caused *pretty severe uterine pains*—as she expressed herself, "*as if she was going to be sick,*" (i. e. be confined;) after taking the third pill, the discharge was "*a mere show.*" Treatment—to take the remaining three pills, one every two hours.

*July 10.* Slept well; pain in the back gone; *the discharge entirely ceased;* feels well; pulse natural; appetite good; skin cool; the uterus contracted and free from pain, on examination per vaginam. Treatment—perfect rest, nourishing diet and cold drinks.

*Vesper.* Improving; *no return* of the discharge.

---

* The Extract of Monesia can be obtained at Mr. Frederick Brown's drug store, North East corner of Fifth and Chestnut streets. For an account of the Monesia, vide Medical Examiner, vol. III., Nos. 13, 14, March 28, and April 4, 1840, pp. 207, 215.

*July* 11. Still *no return* of the hæmorrhage; gaining strength; to take tonics, &c.

*July* 12. Appetite indifferent; *no return.* ℞. Pulv. Colombæ, Pulv. Sub-Carb. Ferri, Rhei, Zingiberis āā ʒj. M. ft. chart. No. xii. S. one three times a-day in molasses. Infusion of Prunus Virginiana, a wine-glassful three times a-day. Diet—oysters, &c.

*Vesper.* Has had, owing to the exertion required in changing her bedding, some slight discharge; checked by one or two pills of the *monesia*, every four hours.

*July* 16. Still using tonics; no pain; *no return of the hæmorrhage;* feels well; anxious to get out of bed.

Supposing that the above case may be interesting as showing, that in the *Extract of Monesia* we have a substance capable of causing powerful uterine contractions, even in small doses, and of arresting profuse menorrhagia of long duration, after the exhibition of all the usual remedies had failed, even when pushed to the extreme; and trusting that this hasty outline of the *effects of monesia* in this, to me, troublesome (although not uninteresting) case, may be of some interest to members of the profession generally,

I remain, gentlemen,
Your obedient servant,
A. D. Chaloner, M. D.

*Philadelphia*, July 10, 1840.

